# Relationship between circulating tumor cells and tumor response in colorectal cancer patients treated with chemotherapy: a meta-analysis

**DOI:** 10.1186/1471-2407-14-976

**Published:** 2014-12-18

**Authors:** Xuanzhang Huang, Peng Gao, Yongxi Song, Jingxu Sun, Xiaowan Chen, Junhua Zhao, Jing Liu, Huimian Xu, Zhenning Wang

**Affiliations:** Department of Surgical Oncology and General Surgery, First Hospital of China Medical University, 155 North Nanjing Street, Heping District, Shenyang City, 110001 PR China

**Keywords:** Circulating tumor cells, Colorectal cancer, Chemotherapy, Tumor response, Prognosis

## Abstract

**Background:**

The prognostic value of circulating tumor cells (CTCs) in colorectal cancer (CRC) patients and their value in predicting tumor response to chemotherapy are controversial. The aim of this meta-analysis was to assess the prognostic and predictive value of CTCs in CRC patients treated with chemotherapy.

**Methods:**

A comprehensive literature search for relevant studies was conducted in PubMed, Embase, the Cochrane Database, the Science Citation Index and the Ovid Database, and the reference lists of relevant studies were also perused for other relevant studies (up to April, 2014). Using the random-effects model in Stata software, version 12.0, the meta-analysis was performed using odds ratios (ORs), risk ratios (RRs), hazard ratios (HRs) and 95% confidence intervals (CIs) as effect measures. Subgroup and sensitivity analyses were also performed.

**Results:**

Thirteen eligible studies were included. Our meta-analysis indicated that the disease control rate was significantly higher in CRC patients with CTC-low compared with CTC-high (RR = 1.354, 95% CI [1.002–1.830], p = 0.048). CRC patients in the CTC-high group were significantly associated with poor progression-free survival (PFS; HR = 2.500, 95% CI [1.746–3.580], p < 0.001) and poor overall survival (OS; HR = 2.856, 95% CI [1.959–4.164], p < 0.001). Patients who converted from CTC-low to CTC-high or who were persistently CTC-high had a worse disease progression (OR = 27.088, 95% CI [4.960–147.919], p < 0.001), PFS (HR = 2.095, 95% CI [1.105–3.969], p = 0.023) and OS (HR = 3.604, 95% CI [2.096–6.197], p < 0.001) than patients who converted from CTC-high to CTC-low.

**Conclusions:**

Our meta-analysis indicates that CTCs are associated with prognosis in CRC patients treated with chemotherapy. Moreover, CTCs could provide additional prognostic information to tumor radiographic imaging and might be used as a surrogate and novel predictive marker for the response to chemotherapy.

**Electronic supplementary material:**

The online version of this article (doi:10.1186/1471-2407-14-976) contains supplementary material, which is available to authorized users.

## Background

Colorectal cancer (CRC) is the third most commonly diagnosed cancer in males and the second in females worldwide [1]. Approximately 50% of CRC patients will develop subsequent metastasis or recurrence, regardless of curative resection. Despite these outcomes, standard combined chemotherapy has been successfully used to increase the cure rate [2, 3]. In recent decades, significant improvements have been made in the response rate, disease control rate, progression-free survival (PFS) and overall survival (OS) of CRC patients [4, 5].

However, despite the improved efficacy of chemotherapy, only a fraction of patients respond to it [6, 7]. Furthermore, there are a lack of accurate markers for predicting tumor response that can be used to identify those patients who might safely discontinue prolonged treatment and those who should resume chemotherapy quickly. Such markers could reduce the use of chemotherapy in nonresponsive patients, reducing unnecessary costs and toxicity [8, 9].

Circulating tumor cells (CTCs) have been detected in the peripheral blood of patients with various cancers [10–12]. Several studies have reported that CTCs can be used as prognostic and predictive markers in patients with breast or prostate cancer [10, 12]. However, the clinical significance of CTCs in CRC patients treated with chemotherapy and targeted agents has not yet been confirmed consistently, and whether CTCs can be used as a predictive marker for response to chemotherapy is controversial.

The aim of our study was to use a meta-analysis to comprehensively summarize the prognostic and predictive significance of CTCs in evaluating the response to chemotherapy in CRC patients.

## Methods

### Search strategy

PubMed, Embase, the Science Citation Index, Cochrane Database and the Ovid Database were systematically searched for studies of the prognostic and predictive significance of CTCs in CRC patients treated with chemotherapy, with no restrictions on language, place of publication or date of publication (up to April, 2014). The reference lists of the retrieved studies and reviews were also perused manually to check for potentially relevant studies. The main search terms used were “circulating tumor cells”, “isolated tumor cells”, “occult tumor cells”, “peripheral blood”, “colorectal cancer”, “colon cancer”, “rectal cancer”, “gastrointestinal cancer”, “chemotherapy” and “targeted treatment/agent”.

### Study eligibility criteria

Studies were considered eligible if they met all of the following criteria: (1) all enrolled patients (>20) were diagnosed with CRC; (2) prognostic and predictive significance of CTCs in patients treated with chemotherapy was assessed with at least one of the outcome measures of interest reported in the study or calculated from the published data; (3) tumor response to chemotherapy was assessed according to the Response Evaluation Criteria In Solid Tumors (RECIST) guidelines (complete response [CR], partial response [PR], stable disease [SD] or progressive disease [PD]) [13]; and (4) the samples were collected from peripheral blood. When several studies were based on the same patient population, only the most informative study was included.

### Data extraction and quality assessment

Two reviewers (X. Z. Huang and P. Gao) independently extracted data from eligible studies. The following information was extracted: first author, year of publication, population characteristics, chemotherapy, sampling times (before the initiation of surgery and chemotherapy [“baseline”] or after the initiation of chemotherapy [“during chemotherapy”]), detection method, rate of CTC positivity, follow-up period, cut-off point, prognostic values (OS and PFS) and objective response to chemotherapy (CR, PR, SD or PD).

The quality of the included studies was assessed with the Newcastle-Ottawa Scale (NOS) criteria for cohort studies [14]. A funnel plot was used to assess publication bias. Any disagreements on data extraction and quality assessment of the included studies were resolved through comprehensive discussion. All written informed consents for participants have been described and obtained by the studies that were included in our meta-analysis.

### Statistical analysis

Our meta-analysis was performed with Stata software, version 12.0 (2011) (Stata Corp, College Station, TX, USA), in accordance with the recommendations of the Preferred Reporting Items for Systematic Reviews and Meta-analyses (PRISMA) [15]. The odds ratios (ORs), risk ratios (RRs) and hazard ratios (HRs) were regarded as effect measures for summarizing prognosis and objective response to chemotherapy. If the HR and its 95% confidence interval (95% CI) were not provided directly, they were calculated from the available data using the method reported by Jayne F. Tierney [16].

To retain maximum information, if one study reported several results separately for different blood samples collected during chemotherapy, we combined multiple effect values into a pooled estimate for further meta-analysis. All relevant studies were included in the overall analysis. A subgroup analysis was performed simultaneously based on the sampling time (baseline or during chemotherapy) and CTC detection method. We assessed the correlation between the CTC level and the response to chemotherapy, assuming radiographic imaging to be the gold standard, and in this way, determined the sensitivity and specificity of CTC-high status in predicting the response rate (CR + PR) or the disease control rate (CR + PR + SD). Sensitivity, specificity, positive predictive value, negative predictive value and area under the receiver operating characteristic curve [ROC] were calculated using the binomial rendition of the bivariate mixed-effects regression model [17, 18].

A p value <0.05 was considered statistically significant and CI was set at 95%. In addition to the diagnostic accuracy test, the remaining analyses used a random-effects model, because it provided more conservative estimates and was more tailored to multicenter studies in which heterogeneity was usually present [19]. Heterogeneity among studies was evaluated using the Cochran Q test and I^2^ statistic [20]. Potential sources of heterogeneity were explored using a meta-regression analysis and Galbraith plot. To maintain the accuracy of the results of the meta-regression, the analysis was only conducted when the number of studies was greater than 10 [21, 22]. We used Egger’s and Begg’s tests to investigate publication bias [23, 24] and conducted trim-and-fill analysis to evaluate the effect of publication bias [25]. A sensitivity analysis was conducted to assess the quality and consistency of results using the leave-one-out approach.

### Ethics statement

The study did not violate the local regulations, and was approved by the Research Ethics Committee of China Medical University, China.

## Results

### Baseline characteristics of the included studies

**Figure 1:**
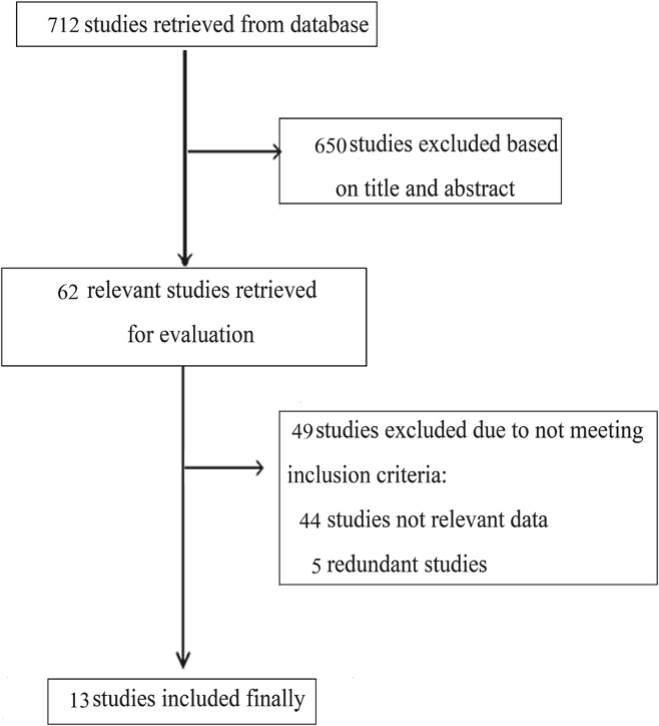
**Selection of studies.** Flow chart showing the selection process for the included studies.

Seven hundred and twelve relevant studies were initially identified in the literature search. After screening titles and abstracts, 650 studies were excluded and 62 potential studies were reviewed further. An additional 49 studies were then excluded because they were redundant studies or lacked the outcomes of interest. Finally, 13 studies were identified as eligible for inclusion in the meta-analysis (Figure 1).

The 13 studies included 2388 eligible CRC patients (median sample size: 76 [38–735]; mean: 184). The studies were conducted in Asia, Europe, Oceania and North America, and were published between 2008 and 2014. In terms of sampling time, seven studies assessed the significance of CTCs detection at baseline and during chemotherapy separately [11, 26–31]: one study assessed the significance of CTCs combined at both time points [32], three assessed the significance of CTC detection only at baseline [33–35], one study assessed the significance of CTC detection only during chemotherapy [36] and one study did not report sampling times [37]. Six studies showed the relationship between clinical outcomes and CTC changes during chemotherapy [11, 26–29, 32]. Nine of the 13 studies only enrolled eligible patients with metastatic CRC (mCRC) [11, 26, 28–31, 33, 34, 37]. Table 1 summarizes the main baseline characteristics and study design variables. The quality of the included studies was evaluated with the NOS and is summarized in Table 2.

**Table 1:** **Baseline characteristics and design variables of the included studies**

Reference	Number (M/F)	C/R/RS	ST	CRT before and after ST	Age mean ± SD/ median (range)	Methods	Rate(+)	Follow up mean ± SD/median (range)	Tumor stage	HR (method no.)	OM	Surgery
Barbazan 2014 [30]	50(37/13)	34/14	baseline	NO/YES	64.5 ± 10.3; 31-84	RT-PCR	13/50	0-40	mCRC	PFS,OS(3)	PFS,OS,	NR
			4 W; 16 W	YES/YES	64.5 ± 10.3; 31-84	RT-PCR	12/50; 15/50	0-40	mCRC	PFS,OS(3); PFS,OS(11)	PFS,OS, RECIST	NR
Kuboki 2013 [34]	63(34/29)	41/22/0	baseline	NO/YES	61(33–81)	Cellsearch	19/63	median:8.7	mCRC	PFS,OS(3)	PFS,OS, RECIST	NR
Lu 2013 [36]	90(51/39)	90/0/0	1,4 W	YES/YES	63.1 ± 12.9	membrane-array	21/90	36(18–61)	III stage	PFS,OS(3)	PFS,OS	YES
Iinuma(1) 2013 [35]	Training: 420(224/196)	NR	baseline	NO/YES	66.0 ± 12.4	RT-PCR	57/150	36.9 ± 19.5	Dukes’ C	PFS,OS(3)	PFS,OS	YES
Iinuma(2) 2013 [35]	Validation: 315(175/140)	NR	baseline	NO/YES	67.5 ± 11.8	RT-PCR	35/97	37.1 ± 18.1	Dukes’ C	PFS,OS(3)	PFS,OS	YES
Sastre 2012 [26]	180(118/62)	121/40/19	baseline	NO/YES	65(40–82)	Cellsearch	85/180	29(0–53.2)	mCRC	PFS,OS(3)	PFS,OS, RECIST	123YES
	147	NR	cycle3	YES/YES	NR	Cellsearch	23/147	NR	mCRC	PFS,OS(3)	PFS,OS, RECIST	123YES
de Albuquerque 2012 [27]	60(43/17)	NR	baseline	NO/YES	65.2(40–80)	RT-PCR	23/33	8.1(6.4-9.7)	II-IV stage	PFS(10)	PFS, RECIST	NR
	33	NR	1-4 W; 5-8 W; 9-12 W	YES/YES	NR	RT-PCR	18/33; 19/33; 17/33	NR	NR	NR	RECIST	NR
Matsusaka 2011 [28]	64(31/33)	36/28	baseline	NO/YES	59(18–72)	Cellsearch	12/64	0-36	mCRC	PFS(3);OS(10)	PFS,OS, RECIST	NR
	/	NR	2 W; 8-12 W	YES/YES	NR	Cellsearch	7/63; 4/60	NR	mCRC	PFS(3);OS(10)	PFS,OS, RECIST	NR
Konigsberg 2010 [33]	38(23/15)	NR	mixed	NO/YES	65(44–82)	ICC	23/38	11.7(8.4-14.9)	mCRC	PFS,OS(3)	PFS,OS, RECIST	NR
Yalcin 2010 [32]	93(50/43)	NR	mixed	part NO/YES	56(24–78)	Cellsearch	30/93	6	II-IV stage	NR	RECIST	PART YES
Tol 2010 [29]	467(284/183)	225/122/120	baseline	NO/YES	63(27–83)	Cellsearch	129/451	16.8	mCRC	PFS,OS(3)	PFS,OS, RECIST	NO
	/	NR	1-2 W	YES/YES	NR	Cellsearch	21/368	NR	mCRC	PFS,OS(3)	PFS,OS, RECIST	NR
	/	NR	3-5 W; 6-12 W; 13-20 W	YES/YES	NR	Cellsearch	17/320; 18/336; 16/254	NR	mCRC	PFS,OS(3)	PFS,OS, RECIST	NR
Yen 2009 [37]	76(44/32)	55/21	NR	NR	64(39–83)	membrane-array	30/76	20(4–34)	mCRC	PFS,OS(3)	PFS,OS, RECIST	YES
Cohen 2008 [11]	430(238/192)	292/71/66	baseline	NO/YES	64(22–92)	Cellsearch	107/413	12.6 ± 6.5/11.0(0.8-30.0)	mCRC	PFS,OS(11)	PFS,OS, RECIST	NR
	/	NR	3-5 W	YES/YES	NR	Cellsearch	38/320	NR	mCRC	PFS,OS(11)	PFS,OS, RECIST	NR
	/	NR	1-2 W; 6-12 W; 13-20 W	YES/YES	NR	Cellsearch	41/357; 25/310; 21/193	NR	mCRC	PFS,OS(11)	PFS,OS, RECIST	NR
Staritz 2004 [31]	42	NR	baseline	NO/YES	NR	RT-PCR	19/41	13.7(1.9-25.4)	mCRC	OS(10)	OS	NR
			1 day	YES/YES	NR	RT-PCR	23/40	13.7(1.9-25.4)	mCRC	OS(10)	OS	NR

NOTE, M/F: Male/female; C/R/RS: Colon/rectum/rectosigmoid; ST: Sample time; CRT: Chemoradiotherapy before and after sampling time; SD: Standard deviation; Rate (+): Rate of CTCs positive patients, n/N (%); OM: Outcome measured; Mixed: samples without separation based on sampling time; mCRC: metastatic colorectal cancer; OS: Overall survival; PFS: Progression-free survival; HR: Hazard Ratio; RECIST: Response evaluation criteria in solid tumors; ICC: Immunocytochemistry; RT-PCR: Reverse-transcriptase polymerase chain reaction; NR: Not reported.

**Table 2:** **The assessment of the risk of bias in each cohort study using the Newcastle-Ottawa scale**

Study	Selection (0–4)				Comparability (0–2)		Outcome (0–3)			Total
	REC	SNEC	AE	DO	SC	AF	AO	FU	AFU	
Barbazan 2014 [30]	0	1	1	1	0	0	1	1	0	5
Kuboki 2013 [34]	0	1	1	1	0	0	1	0	0	4
Lu 2013 [36]	1	1	1	1	0	0	1	1	1	7
Iinuma 2013 [35]	1	1	1	1	0	0	1	1	0	6
Sastre 2012 [26]	1	1	1	1	0	0	1	1	0	6
de Albuquerque 2012 [27]	0	1	1	1	0	0	1	0	1	5
Matsusaka 2011 [28]	0	1	1	1	1	0	1	1	0	6
Konigsberg 2010 [33]	0	1	1	1	0	0	1	0	0	4
Yalcin 2010 [32]	0	1	1	1	0	0	1	0	0	4
Tol 2010 [29]	1	1	1	1	0	0	1	1	0	6
Yen 2009 [37]	1	1	1	1	0	0	1	1	0	6
Cohen2008 [11]	0	1	1	1	0	0	1	1	0	5
Staritz 2004 [31]	0	1	1	1	0	0	1	0	1	5

NOTE. REC: representativeness of the exposed cohort; SNEC: selection of the non-exposed cohort; AE: ascertainment of exposure; DO: demonstration that outcome of interest was not present at start of study; SC: study controls for age, sex; AF: study controls for any additional factors (chemoradiotherapy, curative resection); AO: assessment of outcome; FU: follow-up long enough (36 Months) for outcomes to occur; AFU: adequacy of follow-up of cohorts (≥90%). ‘1’ means that the study is satisfied the item, and ‘0’ means the opposite situation.

### Correlation of CTCs with the objective response to chemotherapy

**Figure 2:**
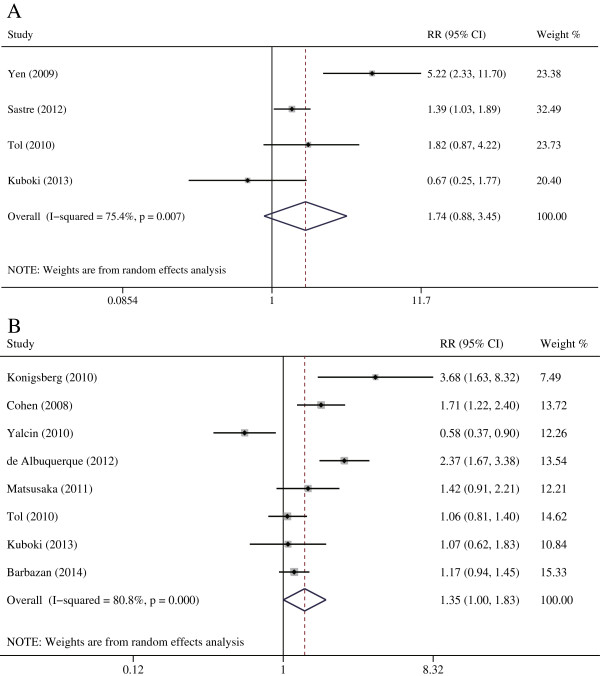
**Risk ratios (RR) summary for correlation of CTCs and tumor response. A**: The risk ratio (RR) was summarized for the correlation of tumor response rate with CTCs detection. **B**: The RR was summarized for the correlation of tumor disease control rate with CTCs detection.

Our meta-analysis suggested that the disease control rate was significantly higher in CRC patients with CTC-low compared with CTC-high (RR = 1.354, 95% CI [1.002–1.830], p = 0.048), and patients with CTC-low tended to have a favorable response rate, although statistical significance was not reached (RR = 1.740, 95% CI [0.878–3.447], p = 0.113) (Figure 2). A similar tendency was obtained in the subgroup analysis based on sampling time: baseline (response rate: RR = 1.258, 95% CI [0.990–1.598], p = 0.060; disease control rate: RR = 1.375, 95% CI [0.961–1.967], p = 0.082) and during chemotherapy (response rate: RR = 2.315, 95% CI [1.242–4.316], p = 0.008; disease control rate: RR = 1.535, 95% CI [1.170–2.016], p = 0.002). After excluding studies containing stage II–III patients, the pooled results of the subgroup analysis of mCRC patients were still significant (disease control rate: RR = 1.354, 95% CI [1.065–1.721], p = 0.013). We also conducted a pooled analysis using the CellSearch system method, a standardized semiautomated quantification method system approved by the U.S. Food and Drug Administration, with similar results. These estimated results for the response to chemotherapy are summarized in Table 3.

**Table 3:** **Detailed results of subgroup analyses for prognostic and predictive significance**

		Sample time		Method		CellSearch	
	Any	Baseline	During-chemotherapy	CellSearch	No-Cellsearch	Baseline	During- chemotherapy
Response rate(RR^i^) ¶^ii^	1.740[0.878-3.447], *I* ^*2*^ = 75.4%	1.258[0.990-1.598], *I* ^*2*^ = 6.9%;Fixed:1.263 [1.008-1.584],*I* ^*2*^ = 6.9%	2.315[1.242-4.316], *I* ^*2*^ = 0%	1.326[0.904-1.946],*I* ^*2*^ = 22.5%	5.2174 [2.3258-11.7041], *N* = 1^iii^	1.258[0.990-1.598], *I* ^*2*^ = 6.9%;Fixed:1.263 [1.008-1.584],*I* ^*2*^ = 6.9%	2.315[1.242-4.316],*I* ^*2*^ = 0%
Disease control rate(RR) Ψ^iv^	1.354[1.002-1.830], *I* ^*2*^ = 80.8%	1.375[0.961-1.967], *I* ^*2*^ = 77.4%	1.535[1.170-2.016],*I* ^*2*^ = 69.8%	1.110[0.784-1.571],*I* ^*2*^ = 74.6%	2.005[1.052-3.821], *I* ^*2*^ = 87.7%	0.974[0.933-1.016], *I* ^*2*^ = 0.0%	1.451[1.116-1.887],*I* ^*2*^ = 36.3%
Disease control rate(RR) ¶	1.354[1.065-1.721], I^2^ = 59.5%	1.248[0.874-1.783], *I* ^*2*^ = 74.7%	1.331[1.099-1.612], *I* ^*2*^ = 35.9%	1.292[1.005-1.660], *I* ^*2*^ = 42.4%	1.933[0.633-5.902],*I* ^*2*^ = 86.0%	0.974[0.933-1.016], *I* ^*2*^ = 0.0%	1.451[1.116-1.887],*I* ^*2*^ = 36.3%
**CTCs change**							
Disease control rate (OR^v^: increase VS decrease) Ψ	27.088[4.960- 147.919], *I* ^*2*^ = 0.0%	/	/	39.000[2.932- 518.841], *N* = 1	20.570[2.170-194.950], *N* = 1	/	/
HR^vi^ for PFS^vii^(L/H to H VS H to L^viii^) ¶	2.095[1.105-3.969], *I* ^*2*^ = 74%	/	/	2.095[1.105-3.969], *I* ^*2*^ = 74%	/	/	/
HR for OS(L/H to H VS H to L) ¶	3.604[2.096-6.197], *I* ^*2*^ = 52.9%	/	/	3.604[2.096-6.197], *I* ^*2*^ = 52.9%	/	/	/
**Prognosis**							
HR for PFS Ψ	2.500[1.746-3.580], *I* ^*2*^ = 90.0%; Without Matsusaka 2.654[2.069-3.405], *I* ^*2*^ = 58.1%	1.853[1.405-2.445], *I* ^*2*^ = 83.6%; Without Matsusaka 1.942[1.549-2.435], *I* ^*2*^ = 47.6%	2.386[1.454-3.917],*I* ^*2*^ = 93.3%; Without Matsusaka 2.694[2.085-3.481], *I* ^*2*^ = 35.1%	1.769[1.180-2.651],*I* ^*2*^ = 90.2%; Without Matsusaka 2.059[1.563-2.712], *I* ^*2*^ = 50.4%	3.387[2.479-4.628],*I* ^*2*^ = 32.7%	1.422[1.102-1.835], *I* ^*2*^ = 82.5%; Without Matsusaka 1.578[1.355-1.837], *I* ^*2*^ = 0%	2.127[1.190-3.802], *I* ^*2*^ = 95%; Without Matsusaka 2.697[2.208-3.294], *I* ^*2*^ = 0%
HR for PFS	2.237[1.485-3.370], *I* ^*2*^ = 91.8%; Without Matsusaka 2.476[1.830-3.350], *I* ^*2*^ = 68.0%	1.621[1.224-2.146], *I* ^*2*^ = 83.6% Without Matsusaka 1.736[1.383-2.180], *I* ^*2*^ = 44.5%	2.100[1.273-3.465],*I* ^*2*^ = 93.7%; Without Matsusaka 2.593[2.152-3.124], *I* ^*2*^ = 0.0%	1.769[1.180-2.651],*I* ^*2*^ = 90.2%; Without Matsusaka 2.059[1.563-2.712], *I* ^*2*^ = 50.4%	3.618[1.976-6.624],*I* ^*2*^ = 65.8%	1.422[1.102-1.835], *I* ^*2*^ = 82.5%; Without Matsusaka 1.578[1.355-1.837], *I* ^*2*^ = 0%	2.127[1.190-3.802], *I* ^*2*^ = 95%; Without Matsusaka 2.697[2.208-3.294], *I* ^*2*^ = 0%
Operation: HR for PFS¶	3.433[2.111-5.583],*I* ^*2*^ = 72.8%	2.377[1.692-3.339], *I* ^*2*^ = 18.8%	3.344[1.135-9.852], *I* ^*2*^ = 75.6%	1.981[1.478-2.654], *N* = 1	4.265[3.079-5.907],*I* ^*2*^ = 0.0%	1.943[1.362-2.770], *N* = 1	2.063[1.231-3.458], *N* = 1
HR for OS^ix^¶	2.856[1.959-4.164],*I* ^*2*^ = 83.4%; Without Matsusaka 3.139[2.167-4.545],*I* ^*2*^ = 76.1%	2.356[1.957-2.836], *I* ^*2*^ = 14.0%; Without Matsusaka 2.288[1.941-2.697], *I* ^*2*^ = 1.7%	3.292[1.611-6.726],*I* ^*2*^ = 96.2%; Without Matsusaka 3.998[2.478-6.449], *I* ^*2*^ = 79.5%	2.452[1.484-4.050],*I* ^*2*^ = 89.9%; Without Matsusaka 2.895[1.697-4.940], *I* ^*2*^ = 85.9%	3.367[1.882-6.023],*I* ^*2*^ = 68.6%	2.310[1.759-3.035], *I* ^*2*^ = 49.9%; Without Matsusaka 2.210[1.716-2.846], *I* ^*2*^ = 46.8%	2.802[1.175-6.684],*I* ^*2*^ = 97.4%; Without Matsusaka 3.792[2.052-7.009],*I* ^*2*^ = 86.5%
HR for OS¶	2.642[1.716-4.067],*I* ^*2*^ = 86.2%; Without Matsusaka 2.968[1.913-4.606],*I* ^*2*^ = 80.4%	2.373[1.873-3.006], *I* ^*2*^ = 34.3%; Without Matsusaka 2.284[1.836-2.842], *I* ^*2*^ = 27.6%	2.787[1.321-5.882],*I* ^*2*^ = 96.6%; Without Matsusaka 3.536[2.149-5.818],*I* ^*2*^ = 81.8%	2.452[1.484-4.050], *I* ^*2*^ = 89.9%; Without Matsusaka 2.895[1.697-4.940],*I* ^*2*^ = 85.9%	2.969[1.053-8.375],*I* ^*2*^ = 78.2%	2.310[1.759-3.035], *I* ^*2*^ = 49.9%; Without Matsusaka 2.210[1.716-2.846], *I* ^*2*^ = 46.8%	2.802[1.175-6.684],*I* ^*2*^ = 97.4%; Without Matsusaka 3.792[2.052-7.009],*I* ^*2*^ = 86.5%
Operation: HR for OS¶	3.681[1.833-7.394],*I* ^*2*^ = 81.8%	1.890[1.407-2.539], *I* ^*2*^ = 0.0%	4.069[0.872-18.980],*I* ^*2*^ = 87.0%	1.738[1.296-2.332], *N* = 1	4.678[2.267-9.654],*I* ^*2*^ = 67.5%	1.642[1.149-2.345], *N* = 1	1.961[1.168-3.295], *N* = 1

^i.^RR: relative risk.

^ii^¶: included patients with metastatic colorectal cancer.

^iii^N = 1: only one eligible study without pooled analysis.

^iv^Ψ: included patients with metastatic and II–IV stages colorectal cancer.

^v^OR: odds ratio.

^vi^HR: hazard ratio.

^vii^PFS: progression-free survival.

^viii^H: high CTCs level; L: low CTCs level.

^ix^OS: overall survival.

For CTCs detected at baseline, sensitivity and specificity of CTC-high for imaging-based disease progression were 52.0% (95% CI [21.5–81.1%]) and 68.7% (95% CI [57.4–78.2%]), respectively. Positive and negative predictive values were 40.4% (95% CI [11.7–77.6%]) and 77.3% (95% CI [46.1–93.1%]), respectively, and the area under the ROC curve was 0.68 (95% CI [0.10–0.98]). Sensitivity and specificity of the CTCs detected during chemotherapy were 50.0% (95% CI [19.6–80.4%]) and 89.5% (95% CI [79.3–95.0%]), respectively. Positive and negative predictive values were 40.9% (95% CI [22.7–62.1%]) and 90.6% (95% CI [80.1–95.8%]), respectively, and the area under the ROC curve was 0.86 (95% CI [0.15–1.00]). For the imaging-based nonresponse (SD + PD), sensitivity and specificity of CTCs detected at baseline and during chemotherapy were 30.2% (95% CI [10.1–62.5%]) and 91.1% (95% CI [75.6–97.1%]), respectively. Positive and negative predictive values were 79.4% (95% CI [69.1–86.9%]) and 50.4% (95% CI [23.9–76.7%]), respectively, and the area under the ROC curve was 0.77 (95% CI [0.73–0.81]).

**Figure 3:**
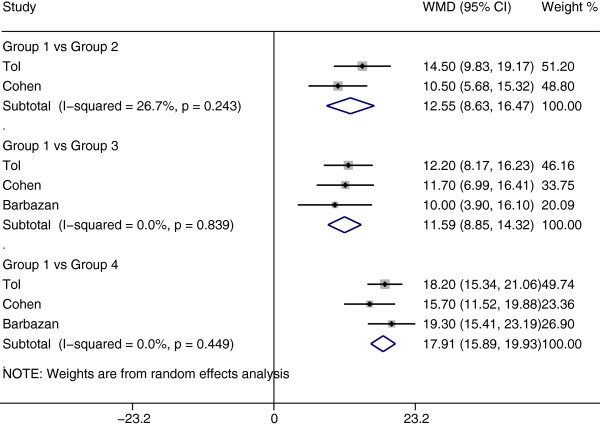
**Results for the meta-analysis of overall survival (OS) time.** The differences in median OS time based on the combination of CTCs and radiographic imaging, Group 1: patients with CTC-low during chemotherapy and disease control, Group 2: patients with CTC-low during chemotherapy and progression disease (PD), Group 3: patients with CTC-high during chemotherapy and disease control, Group 4: patients with CTC-high during chemotherapy and PD.

We analyzed three studies that suggested that CTC levels during chemotherapy could provide additional predictive information on survival outcomes when added to radiographic imaging [11, 29, 30]. The OS of CTC-low patients with disease control (median time: 21.694 months, 95% CI [18.871–24.517]) was significantly longer than that of CTC-low patients with PD (median time: 7.943 months, 95% CI [5.680–10.206]), CTC-high patients with disease control (median time: 9.613 months, 95% CI [6.090–13.137]), and CTC-high patients with PD (median time: 3.327 months, 95% CI [2.398–4.257]). Differences in median OS between CTC-low patients with disease control and patients in the other three groups were statistically significant (p < 0.0001 for all three comparisons) (Figure 3).

### Conversion of CTC levels and prognosis

**Figure 4:**
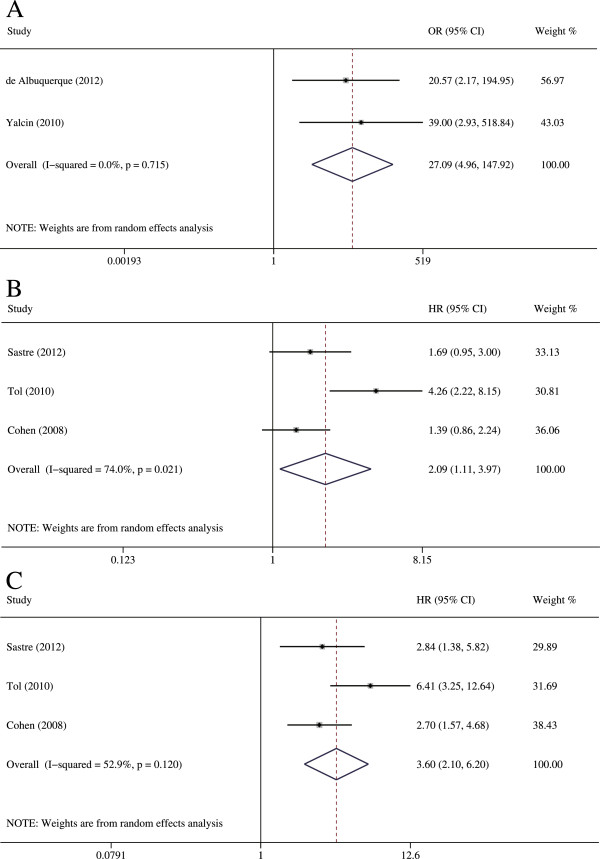
**The results for the relationship between CTCs conversion and prognosis. A**: The estimated odds ratio (OR) was summarized for the correlation of disease control rate with CTCs conversion. **B**: The estimated hazard ratio (HR) was summarized for progression-free survival (PFS) with CTCs conversion. **C**: The estimated HR was summarized for overall survival (OS) with CTCs conversion.

When we compared CTC levels of samples collected at baseline with those collected during chemotherapy, our results suggested that CRC patients who converted from CTC-low to CTC-high or who were persistently CTC-high during treatment had an unfavorable progressive disease compared with those CRC patients who converted from CTC-high to CTC-low (OR = 27.088, 95% CI [4.960–147.919], p < 0.001) (Figure 4). Therefore, it was understandable that CRC patients who became CTC-high or were persistently CTC-high were significantly associated with a poor PFS (HR = 2.095, 95% CI [1.105–3.969], p = 0.023) and a poor OS (HR = 3.604, 95% CI [2.096–6.197], p < 0.001). These results are summarized in Table 3.

### Impact of CTCs on survival outcomes (PFS and OS)

**Figure 5:**
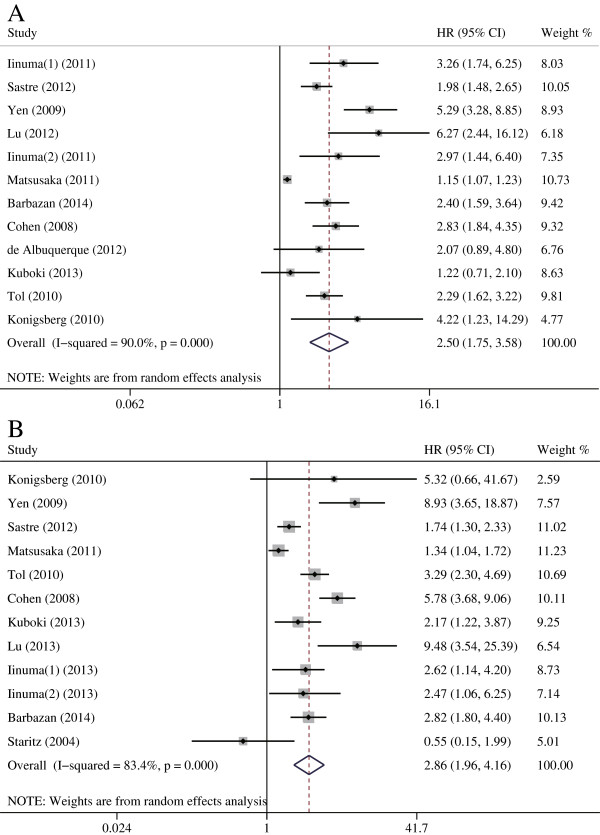
**Estimated hazard ratios (HR) summary for PFS (A) and OS (B). A**: The estimated hazard ratio (HR) was summarized for progression-free survival with CTCs detection. **B**: The estimated HR was summarized for overall survival with CTCs detection.

HRs for PFS were available in eleven studies. The estimated pooled HRs indicated that CTC-high status was associated with a significantly decreased PFS (HR = 2.500, 95% CI [1.746–3.580], p < 0.001). Eleven studies provided HRs on OS and the pooled results indicated that CRC patients in the CTC-high group were significantly associated with a poor OS (HR = 2.856, 95% CI [1.959–4.164], p < 0.001; Figure 5). The pooled results of the subgroup analysis of mCRC patients were similar to the results of the overall analysis when we excluded the four studies containing stage II–III patients (PFS: HR = 2.237, 95% CI [1.485–3.370], p < 0.001; OS: HR = 2.642, 95% CI [1.716–4.067], p < 0.001). Interestingly, for the stage II–III subpopulation, CTCs were still a significant poor prognostic factor for survival: (PFS: HR = 3.226, 95% CI [2.193–4.745], p < 0.001; OS: HR = 3.735, 95% CI [1.710–8.158], p = 0.001). The estimated HRs for PFS and OS were similar in the subgroup analysis of the studies in which most mCRC patients underwent surgery (Table 3).

As shown in the subgroup analysis based on sampling time, CTCs were confirmed as a significant prognostic factor for survival: at baseline (PFS: HR = 1.853, 95% CI [1.405–2.445], p < 0.001; OS: HR = 2.356, 95% CI [1.957–2.836], p = 0.001) and during chemotherapy (PFS: HR = 2.386, 95% CI [1.454–3.917] p = 0.001; OS: HR = 3.292, 95% CI [1.611–6.726], p = 0.001). A similar trend was observed in the pooled analysis using the CellSearch system method (PFS: HR = 1.769, 95% CI [1.180–2.651], p = 0.006; OS: HR = 2.452, 95% CI [1.484–4.050], p < 0.001).

### Evaluation of heterogeneity and publication bias

Neither the direction nor the magnitude of the estimated pooled results was obviously affected when each study was removed in the sensitivity analysis, indicating that no single study dominated our results (Additional file 1). The Galbraith plot showed that the study by Matsusaka et al. [28] contributed substantial heterogeneity to our meta-analysis. Exclusion of the study by Matsusaka et al. [28] could increase statistical power and reduce heterogeneity in the overall and subgroup analyses (Table 3). Begg’s and Egger’s tests were used to examine publication bias (Additional file 2). No significant publication bias was found in the results, except in the HRs for PFS. The trim-and-fill analysis indicated that there might be three unpublished or missing studies existing in the meta-analysis on PFS and the three studies might have an effect on the outcome, and the relationship of CTCs and PFS was still statistically significant if the three studies were published (HR = 2.069, 95% CI [1.505–2.842], p < 0.001). To explore the potential sources of heterogeneity, we conducted a meta-regression that considered the covariates of year, sample size, sampling time and detection method. In a univariate analysis, the explanatory variable that influenced the estimated HRs for PFS and OS was detection method (PFS: coefficient = 0.415, standard error = 0.104, p = 0.001; OS: coefficient = 0.341, standard error = 0.157, p = 0.046), while sampling time was also another potential source of heterogeneity (Additional file 3).

## Discussion

In recent years, standard combined chemotherapy has been widely used for patients with operable or inoperable CRC, and the clinical prognosis of CRC patients has improved [3]. Unfortunately, chemotherapy does not always translate into survival benefits, and unnecessary toxicity can affect patient quality of life [38]. To date, there are no available predictive markers to resolve this problem. Recent studies have assessed whether CTCs can be used as a predictive marker for the response to chemotherapy, but the predictive role of CTCs is still controversial [28].

To our knowledge, this is the first meta-analysis to assess the prognostic and predictive value of CTCs in CRC patients treated with chemotherapy. Our meta-analysis indicated that CTCs detected at baseline and during chemotherapy was significantly associated with prognosis (PFS and OS) in CRC patients. Moreover, for the response to chemotherapy, CTCs detected during chemotherapy were significantly associated with response rate and disease control rate (p < 0.05), and CTCs detected at baseline trended towards an unfavorable response rate and disease control rate, although statistical significance was not reached. A sensitivity analysis, performed by omitting each study individually, confirmed the stability of our results. We also performed a pooled analysis to assess the clinical value of CTCs in mCRC patients only and in stage-II–III patients only, with similar results. These results were consistent with a previous meta-analysis, which suggested that CTC-high patients had unfavorable outcomes [39].

Increasing attention has been paid to the use of CTCs in predicting the efficacy of chemotherapy. Cristofanilli et al. reported that CTC levels could predict the efficacy of chemotherapy in patients with breast cancer earlier than traditional diagnostic methods (at 3–4 weeks versus 9–12 weeks, respectively) [10]. The time at which CTCs were first sampled (range: 1–5 weeks) was generally earlier than the time at which radiographic imaging was performed to assess tumor response (range: 6–25 weeks) in the included studies, when the authors assessed the correlation between CTCs and the response to chemotherapy.

Our results also suggested that the presence of CTCs before and during chemotherapy could be used as an early predictive marker of tumor response in CRC patients treated with chemotherapy. At 1–5 weeks after the initiation of chemotherapy, CTC-high patients had approximately 1.5 times the risk of radiographic PD than CTC-low patients. This could provide earlier opportunities for intervention or for the adjustment of chemotherapy by changing the chemotherapeutic regimen, intensity and/or period. Therefore, CTCs could be very valuable in resolving the problem in which an inefficient chemotherapy regimen is administered to a CRC patient for a prolonged period or a potentially effective chemotherapy regimen is interrupted prematurely.

However, we noted that the sensitivity and specificity of CTCs in predicting the tumor response determined by radiographic imaging were slightly limited. The most plausible explanation may be that the effects of targeted chemotherapy within a few CRC patients do not always translate into a change in tumor size. For example, angiogenesis inhibitors and antivascular therapies (i.e., bevacizumab and sorafenib) could cause necrosis and cavitation without tumor shrinkage [40, 41]. This was consistent with the imprecise correlation between the objective response and the survival outcome [42].

Indeed, several studies included in our analysis evaluated these targeted agents (i.e., bevacizumab or cetuximab) in CRC patients. Therefore, CTCs could provide additional relevant information on the efficacy of chemotherapy, especially in the context of the disadvantages of radiographic imaging. Limitations of CTC detection methods may also explain the limited sensitivity and specificity of CTCs in predicting the tumor response (i.e., moderate sensitivity of immunological techniques and low specificity of the RT-PCR technique). Therefore, the integration of CTC detection at baseline and during chemotherapy (or at different “during-chemotherapy” times) may more accurately predict the tumor response. Further studies are required to investigate ways to improve the accuracy of CTCs in predicting the response to chemotherapy and whether a combined analysis of CTCs and radiographic imaging will be more accurate in assessing the prognosis in CRC patients.

Interestingly, we analyzed the effect of CTCs as a predictive biomarker according to different sampling times (at baseline, 6–12 weeks, 6–12 weeks and 13–20 weeks after the initiation of chemotherapy) and the results suggested that the prognostic and predictive significance of CTCs was relevant to CTC sampling time. This finding concurred with the results of the meta-regression analysis suggesting that sampling time was an important factor. This may occur because of the interaction between CTCs and chemotherapeutic drugs. Furthermore, tumor proliferative activity and response to chemotherapeutic agents may also impact CTC status during chemotherapy [43]. However, the underlying reasons for the different effects of CTCs detected before and during chemotherapy were unclear and will need to be investigated in future studies. In the clinic, the relevance of CTCs as a predictive biomarker may have maximal value in a pre-treatment setting. Meanwhile, CTCs detected during chemotherapy may indicate that the drug given was not working and may not necessarily predict benefit from a higher dose (which would be toxic) or a different drug.

Recent studies have also demonstrated that fluctuations in CTC levels before and during chemotherapy were closely associated with the tumor response to chemotherapy and prognosis, and should be of value in individualizing chemotherapy [44]. Our meta-analysis indicated that there was a more pronounced association between the disease control rate/prognosis and changes in CTC levels than between the disease control rate/prognosis and CTC levels at baseline or during chemotherapy alone (Table 3).

This finding was supported by five of the included studies, which provided prognoses or responses to chemotherapy according to changes in CTC levels [11, 26, 27, 29, 32]. The main reason for this result may be that changes in CTC levels better reflect chemotherapeutic sensitivity and tumor proliferative activity [43]. This finding suggested that fluctuations in CTC levels compensated for the limited sensitivity and specificity of CTCs in predicting the tumor response. Therefore, our results indicated that “real-time” CTC levels detected at different times during chemotherapy, combined with radiographic imaging, should be helpful in guiding individual therapeutic decisions.

Heterogeneity was observed among the studies in our meta-analysis. In particular, the study by Matsusaka et al. [28] contributed substantial heterogeneity to our meta-analysis. A possible reason was that the patients included had better Eastern Cooperative Oncology Group (ECOG) performance scores (0–1 versus 0–2, respectively) and a younger median age than those in the other included studies. Excluding the study by Matsusaka et al. [28], heterogeneity could be decreased but not eliminated.

The meta-regression analysis showed that sampling time and detection method were sources of heterogeneity. Indeed, heterogeneity was reduced in the subgroup analysis based on sampling time and detection method, confirming the results of our meta-regression analysis (Table 3). Heterogeneity could have also been caused by differences in patient characteristics (i.e., age, sex and race), biological differences between colon cancer and rectal cancer, and differences in the chemotherapy strategies used. Furthermore, cytogenetically heterogeneous CTC populations could have contributed to the heterogeneity among the studies [45]. The studies included were all nonrandomized trials and cohort studies; therefore, differences in experimental design may have also given rise to heterogeneity.

Several limitations of this meta-analysis must be noted. First, our meta-analysis was not conclusive regarding when CTCs should be evaluated after the initiation of chemotherapy and what levels of CTCs would be useful for clinical prognostication. Second, as a retrospective study, our meta-analysis was based on published data from the studies included. Several studies did not provide HRs directly and we estimated them from the published data [11, 27, 28]. Third, although the meta-regression showed that sampling time and detection method were the sources of heterogeneity, heterogeneity could not be eliminated because of other factors mentioned above (i.e., patient characteristics, chemotherapy strategies and heterogeneous CTC populations). Therefore, the prognostic power of CTCs must be confirmed with large-scale multicenter studies in homogeneous patients. Fourth, we pooled the studies without separating them into their subcohorts of patients with colon cancer and patients with rectal cancer, because no study provided these data separately. Furthermore, most of the studies included did not comprehensively report patient status regarding surgery or neoadjuvant chemoradiotherapy, thus we could not conduct an in-depth subgroup analysis that adjusted for these confounding factors. The limited number of studies that were included in the subgroup and sensitivity analyses would have also influenced the statistical power of our results. Despite these limitations, our meta-analysis is the first study to assess the prognostic and predictive value of CTCs in CRC patients treated with chemotherapy.

## Conclusions

Our meta-analysis indicates that CTCs could be useful as a surrogate marker for the response to chemotherapy providing additional prognostic information to tumor radiographic imaging. CTCs were also associated with the prognosis of CRC patients treated with chemotherapy. As a “liquid biopsy”, CTC detection is a less invasive way to evaluate the prognosis and response to chemotherapy of CRC patients than a biopsy of the primary tumor. Further high-quality, well-designed, large-scale multicenter studies are required to explore whether an individualized therapeutic decision based on CTC levels would improve the prognosis of CRC patients.

## Electronic supplementary material

Additional file 1: Figure S1: Results of sensitivity analysis based on leave-one-out approach. (PDF 568 KB)

Additional file 2: Table S1: The results of publication bias. (PDF 66 KB)

Additional file 3: Table S2: Results of meta-regression analysis exploring source of heterogeneity (univariate analysis). (PDF 60 KB)

## Abbreviations

CIs: Confidence intervals
CR: Complete response
CRC: Colorectal cancer
CTCs: Circulating tumor cells
HR: Hazard ratio
mCRC: Metastatic CRC
NR: Not reported
OR: Odds ratio
OS: Overall survival
PB: Peripheral blood
PD: Progressive disease
PFS: Progression-free survival
PR: Partial response
RECIST: Response evaluation criteria in solid tumors
ROC: Receiver operating characteristic
RR: Relative risk
SD: Stable disease.
