# Supplementary material for: Relationship between circulating tumor cells and tumor response in colorectal cancer patients treated with chemotherapy: a meta-analysis
Source: BMC Cancer. 2014 Dec 18;14:976. doi: 10.1186/1471-2407-14-976 (PMC4302148; doi:10.1186/1471-2407-14-976)

Additional Figure 1. Results of sensitivity analysis based on leave-one-out approach

A: Progression-free survival (PFS) and CTCs detection; B: overall survival (OS) and CTCs detection.

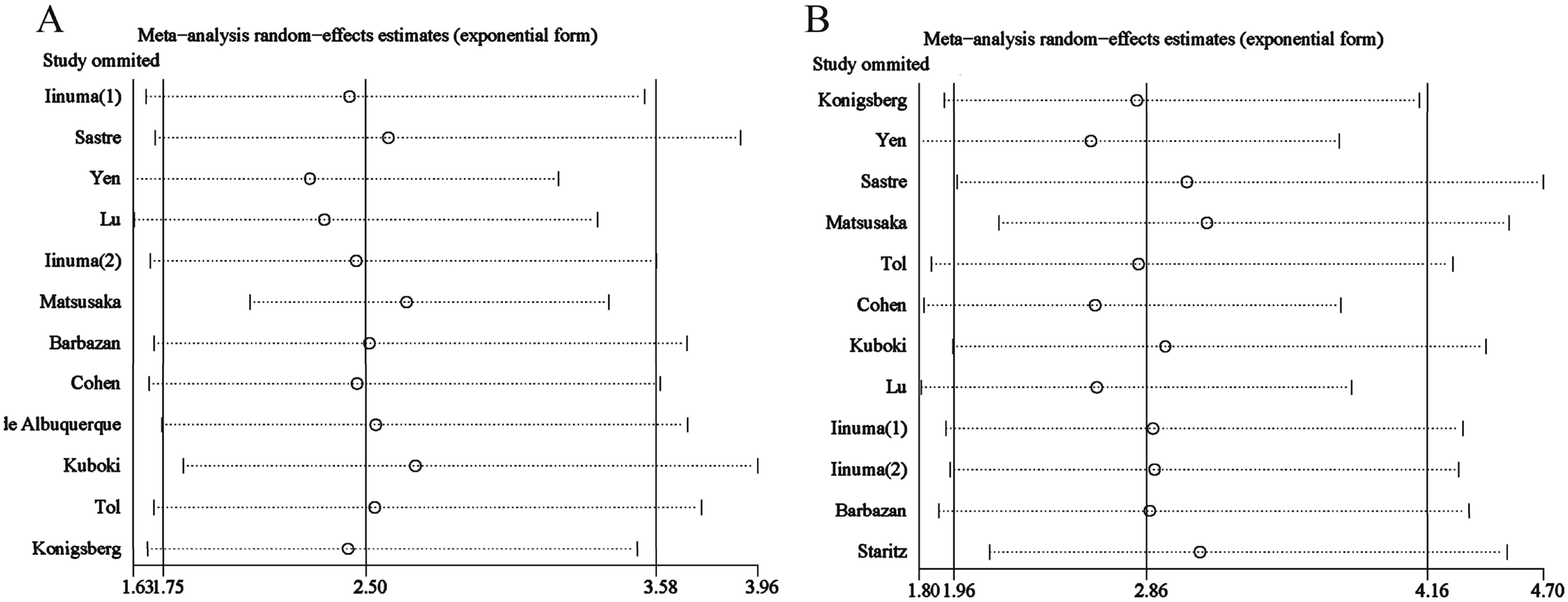

Supplement: Supplementary file 1 — Additional file 1: Figure S1: Results of sensitivity analysis based on leave-one-out approach. (PDF 568 KB) [file 12885_2014_5151_MOESM1_ESM.pdf]
