# Supplementary material for: Relationship between circulating tumor cells and tumor response in colorectal cancer patients treated with chemotherapy: a meta-analysis
Source: BMC Cancer. 2014 Dec 18;14:976. doi: 10.1186/1471-2407-14-976 (PMC4302148; doi:10.1186/1471-2407-14-976)
Supplement: Supplementary file 2 — Additional file 2: Table S1: The results of publication bias. (PDF 66 KB) [file 12885_2014_5151_MOESM2_ESM.pdf]

Additional Table 1. The results of publication bias

Comparison	*P* (Begg's Test, continuity corrected)	*P* (Egger's test)
**Response rate**	1	0.727
**Disease control rate**	0.536	0.548
**HR for OS**	0.631	0.139
**HR for PFS**	1	0.0002
**CTCs change**		
**OS**	1	0.679
**PFS**	0.296	0.277

NOTE. HR: hazard ratio; OS: overall survival; PFS: progression free survival; CTCs: circulating tumor cells
