# Supplementary material for: Relationship between circulating tumor cells and tumor response in colorectal cancer patients treated with chemotherapy: a meta-analysis
Source: BMC Cancer. 2014 Dec 18;14:976. doi: 10.1186/1471-2407-14-976 (PMC4302148; doi:10.1186/1471-2407-14-976)
Supplement: Supplementary file 3 — Additional file 3: Table S2: Results of meta-regression analysis exploring source of heterogeneity (univariate analysis). (PDF 60 KB) [file 12885_2014_5151_MOESM3_ESM.pdf]

Additional Table 2. Results of meta-regression analysis exploring source of heterogeneity (univariate analysis)

	PFS				PFS omitting Matsusaka et al.			
**Covariates**	**Coefficient**	**Standard error**	**P**	**Adjusted R^2**	**Coefficient**	**Standard error**	**P**	**Adjusted R^2**
**Detection method**	0.4146	0.1041	0.001	55.95%	0.3460	0.0932	0.003	64.60%
**Sampling time**	0.3544	0.1889	0.08	19.83%	0.4370	0.1405	0.008	78.03%
**Sample size**	-0.0004	0.0009	0.692	-10.01%	-0.0012	0.0008	0.143	16.46%
**Publication year**	-0.0380	0.0733	0.612	-6.02%	-0.0343	0.0634	0.597	-10.43%
	**OS**				**OS omitting Matsusaka et al.**			
**Detection method**	0.3406	0.1567	0.046	23.53%	0.3146	0.1543	0.062	20.74%
**Sampling time**	0.1217	0.2547	0.64	-3.35%	0.2908	0.2400	0.247	25.53%
**Sample size**	0.0005	0.0012	0.68	-7.76%	0.0002	0.0012	0.849	-15.14%
**Publication year**	0.0082	0.0708	0.909	-10.28%	0.0018	0.0684	0.979	-13.08%

NOTE: The dependent variable is the lnHR for PFS from each study. Weights have been assigned according to the estimated variance of the lnHR. Adjusted R^2: Proportion of between-study variance explained
